# Association of Exposure to High-risk Antibiotics in Acute Care Hospitals With Multidrug-Resistant Organism Burden in Nursing Homes

**DOI:** 10.1001/jamanetworkopen.2021.44959

**Published:** 2022-02-01

**Authors:** Kyle J. Gontjes, Kristen E. Gibson, Bonnie J. Lansing, Julia Mantey, Karen M. Jones, Marco Cassone, Joyce Wang, John P. Mills, Lona Mody, Payal K. Patel

**Affiliations:** 1 Division of Geriatric & Palliative Medicine, Department of Internal Medicine, University of Michigan Medical School, Ann Arbor; 2 Department of Epidemiology, University of Michigan School of Public Health, Ann Arbor; 3 Department of Microbiology & Immunology, University of Michigan Medical School, Ann Arbor; 4 Division of Infectious Diseases, Department of Internal Medicine, University of Michigan Medical School, Ann Arbor; 5 Geriatrics Research Education and Clinical Center, VA Ann Arbor Healthcare System, Ann Arbor, Michigan; 6 Division of Infectious Diseases, Department of Internal Medicine, VA Ann Arbor Healthcare System, Ann Arbor, Michigan

## Abstract

**Importance:**

Little is known about the contribution of hospital antibiotic prescribing to multidrug-resistant organism (MDRO) burden in nursing homes (NHs).

**Objectives:**

To characterize antibiotic exposures across the NH patient’s health care continuum (preceding health care exposure and NH stay) and to investigate whether recent antibiotic exposure is associated with MDRO colonization and room environment contamination at NH study enrollment.

**Design, Setting, and Participants:**

This is a secondary analysis of a prospective cohort study (conducted from 2013-2016) that enrolled NH patients and followed them up for as long as 6 months. The study was conducted in 6 NHs in Michigan among NH patients who were enrolled within 14 days of admission. Clinical metadata abstraction, multi-anatomical site screening, and room environment surveillance for MDROs were conducted at each study visit. Data were analyzed between May 2019 and November 2021.

**Exposures:**

Antibiotic data were abstracted from NH electronic medical records by trained research staff and characterized by class, route, indication, location of therapy initiation, risk for *Clostridioides difficile* infection (C diffogenic agents), and 2019 World Health Organization Access, Watch, and Reserve (AWARE) antibiotic stewardship framework categories.

**Main Outcomes and Measures:**

The primary outcomes were MDRO colonization and MDRO room environment contamination at NH study enrollment, measured using standard microbiology methods. Multivariable logistic regression was used to identify whether antibiotic exposure within 60 days was associated with MDRO burden at NH study enrollment. Additionally, antibiotic exposure data were characterized using descriptive statistics.

**Results:**

A total of 642 patients were included (mean [SD] age, 74.7 [12.2] years; 369 [57.5%] women; 402 [62.6%] White; median [IQR] NH days to enrollment, 6.0 [3.0-7.0]). Of these, 422 (65.7%) received 1191 antibiotic exposures: 368 (57.3%) received 971 hospital-associated prescriptions, and 119 (18.5%) received 198 NH-associated prescriptions. Overall, 283 patients (44.1%) received at least 1 C diffogenic agent, and 322 (50.2%) received at least 1 high-risk WHO AWARE antibiotic (watch or reserve agent). More than half of NH patients (364 [56.7%]) and room environments (437 [68.1%]) had MDRO-positive results at enrollment. In multivariable analysis, recent antibiotic exposure was positively associated with baseline MDRO colonization (odds ratio [OR], 1.70; 95% CI, 1.22-2.38) and MDRO environmental contamination (OR, 1.67; 95% CI, 1.17-2.39). Exploratory stratification by C diffogenic agent exposure increased the effect size (MDRO colonization: OR, 1.99; 95% CI, 1.33-2.96; MDRO environmental contamination: OR, 1.86; 95% CI, 1.24-2.79). Likewise, exploratory stratification by exposure to high-risk WHO AWARE antibiotics increased the effect size (MDRO colonization: OR, 2.32; 95% CI, 1.61-3.36; MDRO environmental contamination: OR, 1.86; 95% CI, 1.26-2.75).

**Conclusions and Relevance:**

The findings of this study suggest that high-risk, hospital-based antibiotics are a potentially high-value target to reduce MDROs in postacute care NHs. This study underscores the potential utility of integrated hospital and NH stewardship programming on regional MDRO epidemiology.

## Key Points

**Question:**

Is hospital antibiotic exposure associated with multidrug-resistant organism (MDRO) colonization and room environment contamination in nursing homes?

**Findings:**

In this secondary analysis of data from 642 patients enrolled in a prospective cohort study, recent antibiotic exposure was positively associated with both MDRO patient colonization and room environment contamination at nursing home study enrollment. The effect size of these associations increased when antibiotic exposure histories were stratified by risk for *Clostridioides difficile* infection and by exposure to high-risk World Health Organization Access, Watch, and Reserve antibiotic stewardship framework categories.

**Meaning:**

The findings suggest that high-risk hospital antibiotic prescribing is a potentially high-value antibiotic stewardship target to reduce MDRO burden in postacute care nursing homes.

## Teaser

This secondary analysis of a cohort study characterizes antibiotic exposures across the nursing home patient’s health care continuum and investigates whether recent antibiotic exposure is associated with multidrug-resistant organism colonization and room environment contamination.

## Introduction

Nursing home (NH) patients are at a heightened risk for antibiotic-associated adverse events due to a convergence of patient-, facility-, and health care system–level risk factors.^1,2^ NH patients are often older and have multimorbidity, functional disability, indwelling devices, and frequent exposure to antibiotics.^1,2^ These patients often traverse multiple health care facilities and are commonly rehospitalized within 30 days.^3,4^ Across this population’s health care continuum, antibiotic exposure is common, as approximately 30% to 50% of hospitalized patients and 40% to 70% of NH patients receive an antibiotic.^5,6,7^ Antibiotic overuse in this population is associated with multidrug-resistant organism (MDRO) colonization and infection, *Clostridioides difficile* infection (CDI), selection for antibiotic resistance, rehospitalization, unnecessary health care expenditures, and mortality.^1,2^

Our understanding of how hospital and NH infection prevention programming and antibiotic prescribing patterns influence regional health care systems is evolving.^8,9,10^ Patient-transfer networks have been implicated in the regional transmission of MDROs.^11,12,13^ Furthermore, genomic epidemiology and network modeling have supported the role of patient characteristics, transfer networks, facility-level antibiotic stewardship practices, and health care system–level factors in regional MDRO prevalence, infection rates, and transmission.^11,12,13,14^

Amid an emerging understanding of the implications of health care system interconnectedness, little is known about the antibiotic exposures of postacute care NH patients and their influence on MDRO epidemiology. Given the contribution of antibiotics to long-term gut dysbiosis,^15^ recent antibiotic exposure has been recognized to increase the risk of MDRO acquisition in NHs.^16,17,18^ Concurrently, antibiotic exposure has been proposed to increase patient shedding of MDROs onto their proximal environment.^19,20,21^ Therefore, we hypothesized that hospital-based antibiotic exposures would be associated with both NH patient MDRO colonization and NH room environment contamination (eFigure 1 in the Supplement).

Using data collected from a prospective, longitudinal cohort study of NH patients,^16^ we characterized each patient’s prestudy enrollment antibiotic exposures (predominantly hospital-associated prescriptions) and their antibiotic exposures during their NH stay, including hospital-associated prescriptions and NH-associated prescriptions. Additionally, we evaluated the association of antibiotic exposure within 60 days of study enrollment with baseline NH MDRO colonization and NH room environment contamination, with a focus on high-risk antibiotic stewardship targets, as defined by heightened risk for CDI (C diffogenic agents)^22,23,24,25,26^ and by selection for antibiotic resistance, according to the World Health Organization’s (WHO) Access Watch and Reserve (AWARE) antibiotic stewardship framework.^27,28^

## Methods

### Parent Study Design

Newly admitted NH patients were enrolled in a prospective, longitudinal cohort study in 6 Michigan NHs between 2013 and 2016.^16^ The parent study conducted MDRO surveillance of patients and their room environment upon enrollment, at days 14 and 30, and monthly thereafter for as long as 6 months or until discharge.^16^ Patients were eligible if they (or their legally authorized representative) provided written informed consent and were enrolled within 14 days of NH admission.^16^ The parent study was approved by the University of Michigan’s institutional review board, and this secondary analysis was covered by the approval of the parent study. Here, we follow the Strengthening the Reporting of Observational Studies in Epidemiology (STROBE) reporting guidelines.

### Clinical Data Collection

Patient characteristics were obtained at study enrollment. Demographic characteristics included the patient’s age, sex, and race. In the parent study, demographic data on patient race (Asian [2 patients], American Indian or Alaska Native [0 patients], Black or African American [238 patients], Native Hawaiian or Pacific Islander [0 patients], and White [402 patients]) and ethnicity (Hispanic or Latino [6 patients] and non-Hispanic or Latino [396 patients]) were collected by trained research personnel from patient-reported information. We analyzed race using a binary variable of White patients (those identifying as White, Hispanic or Latino, and non-Hispanic or Latino) and patients of other races (Asian and African American). Race and ethnicity data were required to be collected and reported by the funding source. Clinical characteristics included the presence of indwelling devices (feeding tube or urinary catheter), Charlson Comorbidity Index (CCI) score,^29^ and the Physical Self-Maintenance Scale (PSMS) score.^30^ To understand a patient’s health care trajectory, we used the Minimum Data Set 3.0 at study enrollment and discharge to identify the patient’s locations before NH arrival, discharge destinations, and readmissions to the NH.^31^

### Antibiotic Exposure Data Collection

Frontline study personnel (K.E.G. and B.J.L.) abstracted antibiotic exposure data from NH medical records on enrollment and each follow-up visit. Antibiotic-associated data of interest included the generic name, documented indication, and route of administration. For this study, antivirals, antifungals, topical antibiotics, and ocular antibiotics were excluded owing to low sample size. Additionally, antibiotic dose and duration were not used owing to missing data.

Antibiotic data were curated and characterized by an infectious disease physician with antibiotic stewardship expertise (P.K.P.) and an epidemiologist (K.J.G.) using a priori knowledge and prior literature. Antibiotics were classified as hospital-associated prescriptions if they were associated with indications diagnosed during the patient’s hospitalization (eFigure 2 in the Supplement).^32,33^ NH prescriptions identified as a continuation of hospital therapy or associated with a hospital indication were also classified as hospital-associated prescriptions, unless identified as a novel new start. Therefore, antibiotics were classified as NH-associated prescriptions if they were initiated during the patient’s NH stay and were (1) for a new indication; (2) not a continuation of therapy for a prior diagnosis; and/or (3) not an oral transition from a previously intravenous antibiotic. Antibiotics prescribed in alternative locations (ie, emergency department, antecedent NH stay, or outpatient clinics) were classified as other.

Antibiotics were further characterized using antibiotic stewardship metrics. We categorized antibiotics into their class and subclass. Antibiotics prescribed for skin and soft tissue infection (SSTIs) were subcategorized into complicated or uncomplicated SSTI.^34^ Furthermore, antibiotics prescribed for SSTIs were classified as broad-spectrum if they held activity against *Pseudomonas aeruginosa*.^35,36^ Anti-anaerobic antibiotics of interest included ertapenem, imipenem, meropenem, doripenem, tigecycline, moxifloxacin, cefoxitin, metronidazole, amoxicillin/clavulanic acid, ampicillin/sulbactam, piperacillin/tazobactam, and clindamycin.^37^ Inappropriate anti-anaerobic therapy (double anaerobic coverage) was defined as the concurrent prescription of 2 anti-anaerobic agents.^38^

Antibiotics were defined as high-risk, C diffogenic agents if they predispose patients to a markedly high-risk of CDI, as per literature. They include fluoroquinolones; third-, fourth-, and fifth-generation cephalosporins; penicillin combinations; lincosamides; and carbapenems.^22,23,24,25,26^ Patients without exposure to a high-risk C diffogenic agent but with exposure to other antibiotics were classified as having a low-risk antibiotic exposure history. Antibiotics were further classified by the WHO’s 2019 antibiotic classification framework Access, Watch, and Reserve (AWARE).^27^ Briefly, *access antibiotics* correspond to common front-line or second-line antibiotics; *watch antibiotics* indicate high-priority antibiotics with toxic effects or antibiotic resistance concerns; and *reserve antibiotics* are generally last-line treatments for MDRO infections.^28^ High-risk antibiotic classifications for study antibiotics are reported in eTable 1 in the Supplement.

### Microbiology Specimen Collection and Processing

In the parent study, enrolled patients had their dominant hand, nares, oropharynx, groin, and perianal area cultured for MDRO colonization at each study visit.^16^ Patient wound, suprapubic catheter site, and enteral feeding tube insertion site specimens were collected when present and accessible. Surveillance of high-touch patient room environment sites was also performed at each visit.^39,40^ Specimen collection and isolation of methicillin-resistant *Staphylococcus aureus* (MRSA), vancomycin-resistant enterococci (VRE), and resistant gram-negative bacilli (R-GNB) were performed using standard microbiology techniques described elsewhere.^16^ Gram-negative bacilli were classified as R-GNB isolates if they were resistant to 1 or more of the following antibiotics: ceftazidime (30 μg), ceftazidime/clavulanic acid (30 μg/10 μg), ciprofloxacin (5 μg), and imipenem (10 μg).^16^

### Statistical Analysis

Descriptive statistics were used to report baseline patient characteristics. To align with our aims, the patient population was stratified by prestudy enrollment antibiotic exposure history. We used χ^2^ tests or Fisher exact tests (as appropriate) and 2-sample *t* tests to test for significant differences between subgroups.

Next, we used descriptive statistics to characterize patient antibiotic exposures. For these analyses, we categorized patient antibiotic exposures as a dichotomous variable. Antibiotics were classified by time (preenrollment or during study participation) and location of therapy initiation (hospital-associated prescriptions or NH-associated prescriptions). To assess whether there were significant differences between hospital- and NH-associated therapies, we compared the characteristics of hospital-associated prescriptions with NH-associated prescriptions using the χ^2^ test. We focused analysis on our prespecified high-level antibiotic stewardship classifications to minimize the identification of spurious associations owing to small sample size (ie, class and indication data).

Next, to assess whether recent antibiotic exposure was associated with NH MDRO outcomes, we used multivariable logistic regression to investigate whether antibiotic exposure within 60 days of study enrollment was associated with patient colonization and room environment contamination on NH study enrollment. These analyses were performed for the presence of any MDRO and separately for each MDRO (MRSA, VRE, and R-GNB).

For each outcome, we used 2 logistic regression model iterations. The initial model used a dichotomous indicator variable for the patient’s antibiotic exposure history, while the second exploratory model iteration used 2 indicator variables: low-risk and high-risk antibiotic exposure history. For each model, the reference group consisted of patients without antibiotic exposure histories. The exploratory analyses were performed separately for our C diffogenic agent classification schema and our high-risk WHO AWARE exposure history classification schema. The WHO AWARE exposure categories were reclassified into a high-risk exposure framework to simplify interpretation and to account for a small sample size. Briefly, patients with exposure to only access agents were classified as having a low-risk exposure history, while patients with exposure to watch or reserve agents as their highest AWARE category exposure were classified as having a high-risk WHO AWARE exposure history. Each regression model was adjusted for age, sex, race (White vs other), PSMS score, CCI score, indwelling device presence on enrollment, hospital stay longer than 14 days, and NH days to enrollment. Variables were included into the model using a priori knowledge and prior literature.^16^ All regression analyses were adjusted for clustering by facility. We used 2-tailed *P* < .05 as the threshold for statistical significance. Data were analyzed between May 2019 and November 2021 using SAS version 9.4 (SAS Institute).

## Results

### Study Group Characteristics

We enrolled 651 patients in the parent study.^16^ A total of 642 patients had nonmissing antibiotic exposure histories and were included in this analysis. The mean age was 74.7 years (SD, 12.2 years), 369 (57.5%) were women, and 402 (62.6%) were White individuals. At study enrollment, the median (IQR) CCI score was 2.0 (1.0-4.0), the median (IQR) PSMS score was 13.0 (11.0-17.0), and 66 patients (10.3%) had an indwelling device. Overall, 609 patients (94.9%) were admitted from an acute care hospital to the NH for postacute care. Of these, 60 (9.9%) had hospital stays longer than 14 days. The remaining patients were admitted for long-term care (29 originated from another NH, and 4 were admitted from home). After right-tail adjustment to 180 days, the median (IQR) NH duration of stay was 28.0 (18.0-50.0) days. The median (IQR) NH days to study enrollment was 6.0 days (3.0-7.0). Patient characteristics stratified by prestudy enrollment antibiotic exposure histories are reported in Table 1.

**Table 1.** Baseline Characteristics of 642 Nursing Home Patients, Stratified by Prestudy Enrollment Antibiotic Exposure History

Characteristic	Patients, No. (%)			*P* value
	All patients (N = 642)	No antibiotic exposure history (n = 269)	History of antibiotic exposure (n = 373)	
Demographics				
Age, mean (SD), y	74.7 (12.2)	75.1 (13.0)	74.4 (11.6)	.46
Female sex	369 (57.5)	156 (58.0)	213 (57.1)	.82
Male sex	273 (42.5)	113 (42.0)	160 (42.9)	
Race				
Black	238 (37.1)	121 (45.0)	117 (31.4)	<.001^a^
White	402 (62.6)	148 (55.0)	254 (68.1)	
Asian	2 (0.3)	0	2 (0.5)	
Clinical characteristics				
Charlson Comorbidity Score, median (IQR)	2.0 (1.0-4.0)	2.0 (1.0-4.0)	2.0 (1.0-4.0)	.89
Physical Self-maintenance Score, median (IQR)	13.0 (11.0-17.0)	13.0 (11.0-17.0)	13.0 (11.0-17.0)	.69
Indwelling device use^b^	66 (10.3)	17 (6.3)	49 (13.1)	.005
Health care trajectory				
Previous hospitalization	609 (94.9)	242 (90.0)	367 (98.4)	<.001
Hospital stay >14 d	60 (9.4)	12 (4.5)	48 (12.9)	<.001
Length of nursing home stay, median (IQR)^c^	28.0 (18.0-50.0)	28.0 (20.0-49.0)	28.0 (16.0-50.0)	.21
Nursing home days to enrollment, median (IQR)	6.0 (3.0-7.0)	6.0 (3.0-7.0)	5.0 (3.0-7.0)	.21

^a^ *P *value from Fisher exact test.

^b^ Indwelling device use was defined as the presence of a feeding tube or indwelling urinary catheter on study enrollment.

^c^ To minimize skew, right tail adjustment of length of stay greater than 180 days was performed.

### Antibiotic Exposures Across the Health Care Continuum

Of 642 patients, 422 (65.7%) received 1191 antibiotics, and 373 patients (58.0%) had an antibiotic exposure within 60 days of study enrollment. When stratifying preenrollment exposures by their location of therapy initiation, 355 (55.3%) received 818 hospital-associated prescriptions and 16 (2.5%) received 17 NH-associated prescriptions. In total, 368 patients (57.3%) received 971 hospital-associated prescriptions, and 119 patients (18.5%) received 198 NH-associated prescriptions across their health care continuum. Additionally, 13 patients (2.0%) received 22 antibiotics that were classified as other, either due to alternative prescribing locations (18 antibiotics) or missing data (4 antibiotics).

Hospital-associated prescriptions and NH-associated prescriptions were categorized by class and stratified by their location of therapy initiation (Table 2). Of the 1191 total antibiotics, cephalosporins were the most common class (353 [29.6%]), followed by glycopeptides (191 [16.0%]), and fluoroquinolones (176 [14.8%]). Hospital-associated prescriptions and NH-associated prescriptions with documented indications were stratified by their location of therapy initiation (Table 3). For antibiotics with documented indications (1021 of 1191 [85.7%]), the most common indications were urinary tract infections (UTIs) (256 [25.1%]), SSTIs (201 [19.7%]), and respiratory tract infections (164 [16.1%]). Of the 170 antibiotics with unknown indications, 159 (93.5%) were classified as hospital-associated prescriptions. Of the 1074 (90.2%) antibiotics with known routes of therapy, 573 (53.4%) were intravenous.

**Table 2.** Antibiotic Exposures Prescribed by Antibiotic Class, Stratified by Location of Therapy Initiation

Antibiotic class	Prescriptions, No. (%)^a^			
	Hospital-associated prescriptions			Nursing home–associated prescriptions (n = 198)
	All (n = 971)	Pre–study enrollment (n = 818)	During study enrollment (n = 153)	
Cephalosporin				
Any	309 (31.8)	265 (32.4)	44 (28.9)	41 (20.7)
First generation	108 (11.1)	100 (85.5)	8 (66.6)	28 (87.5)
Second generation	18 (1.9)	14 (12.0)	4 (33.3)	4 (12.5)
Third generation	115 (11.8)	103 (67.0)	12 (37.5)	6 (66.6)
Fourth generation	65 (6.7)	45 (33.0)	20 (62.5)	3 (33.3)
Fifth generation	3 (0.3)	3 (2.5)	0	0
Glycopeptides				
Any	173 (17.8)	134 (16.4)	39 (25.7)	17 (8.6)
Intravenous vancomycin	122 (12.6)	98 (73.1)	24 (61.5)	6 (35.3)
Oral vancomycin	28 (2.9)	15 (11.2)	13 (33.3)	11 (64.7)
Unknown route	23 (2.4)	21 (15.7)	2 (5.1)	0 (0.0)
Quinolone	121 (12.5)	106 (12.9)	15 (9.9)	52 (26.3)
Penicillin combination	70 (7.2)	57 (7.0)	13 (8.6)	7 (3.5)
Nitroimidazole	58 (6.0)	53 (6.5)	5 (3.3)	15 (7.6)
Sulfonamide	37 (3.8)	31 (3.8)	6 (4.0)	20 (10.1)
Macrolide	38 (3.9)	34 (4.2)	4 (2.6)	17 (8.6)
Lincosamide	36 (3.7)	33 (4.0)	3 (2.0)	4 (2.0)
Penicillin	30 (3.1)	27 (3.3)	3 (2.0)	9 (4.6)
Carbapenem	30 (3.1)	23 (2.8)	7 (4.6)	2 (1.0)
Tetracycline	26 (2.7)	21 (2.6)	3 (2.0)	5 (2.5)
Nitrofuran	6 (0.6)	6 (0.7)	0	8 (4.0)
Lipopeptide	13 (1.3)	10 (1.2)	3 (2.0)	0
Aminoglycoside	12 (1.2)	9 (1.1)	3 (2.0)	0
Oxazolidinone	7 (0.7)	6 (0.7)	1 (0.7)	1 (0.5)
Ansamycin	2 (0.2)	1 (0.1)	1 (0.7)	0 (0.0)
Monobactam	2 (0.2)	2 (0.2)	0	0
Glyglycycline	1 (0.1)	1 (0.1)	0	0

^a^ Antibiotics were classified as hospital-associated prescriptions if they were associated with indications diagnosed during a hospitalization. Nursing home exposures identified as a continuation of hospital therapy or associated with a hospital indication were also classified as hospital-associated, unless identified as a novel new start. Conversely, antibiotics were classified as nursing home–associated prescriptions if they were initiated during the patient’s nursing home stay and were (1) for a new indication; (2) not a continuation of therapy for a prior diagnosis; and/or (3) not an oral transition of a previously intravenous antibiotic.

**Table 3.** Documented Antibiotic Indications, Stratified by Location of Therapy Initiation

Antibiotic indication	Prescriptions, No. (%)^a^			
	Hospital-associated prescriptions			Nursing home–associated prescriptions (n = 192)
	All (n = 812)	Pre–study enrollment (n = 685)	During study enrollment (n = 127)	
UTI	163 (20.1)	140 (20.4)	23 (18.1)	92 (47.9)
SSTI	166 (20.4)	141 (20.6)	25 (19.7)	28 (14.6)
RTI	130 (16.0)	110 (16.1)	20 (15.8)	30 (15.6)
Empirical and sepsis	104 (12.8)	85 (12.4)	19 (15.0)	12 (6.3)
Surgery or prophylaxis	97 (12.0)	96 (14.0)	1 (0.8)	5 (2.6)
CDI	37 (4.6)	24 (3.5)	13 (10.2)	16 (8.3)
Osteomyelitis	23 (2.8)	14 (2.0)	9 (7.1)	0
Bacteremia	15 (1.9)	9 (11.3)	6 (4.7)	0
GI-related, non-CDI	10 (1.2)	10 (1.5)	0	2 (1.0)
Surgical site infection	8 (1.0)	5 (0.7)	3 (2.4)	1 (0.5)
Other	59 (7.3)	51 (7.4)	8 (6.3)	6 (3.1)

Abbreviations: CDI, *Clostridioides difficile* infection; GI, gastrointestinal infection; RTI, respiratory tract infection; SSTI, skin and soft tissue infection; UTI, urinary tract infection.

^a^ Antibiotics were classified as hospital-associated prescriptions if they were associated with indications diagnosed during a hospitalization. Nursing home exposures identified as a continuation of hospital therapy or associated with a hospital indication were also classified as hospital–associated, unless identified as a novel new start. Conversely, antibiotics were classified as nursing home-associated prescriptions if they were initiated during the patient’s nursing home stay and were (1) for a new indication; (2) not a continuation of therapy for a prior diagnosis; and/or (3) not an oral transition of a previously intravenous antibiotic.

We further classified antibiotic exposures using antibiotic stewardship metrics: 38 of 201 SSTI treatments (18.9%) were classified as broad spectrum; 10 (5.0%) were treating complicated SSTIs. Overall, 163 patients (25.4%) received 243 anti-anaerobic antibiotics: there were 5 instances of inappropriate, double anti-anaerobic coverage. When classifying antibiotic exposures by risk for CDI, 283 patients (44.1%) patients received 525 (44.1%) C diffogenic agents. When classifying antibiotic exposures into WHO AWARE framework categories, 100 patients (15.6%) patients received only access antibiotic exposures, 296 (46.1%) received at least 1 watch antibiotic as their highest-risk exposure, and 26 (4.0%) received at least 1 reserve antibiotic. In total, 430 antibiotics (36.1%) were access agents, 733 (61.5%) were watch agents, and 28 (2.4%) were reserve agents. Collectively, 831 (69.8%) antibiotics were classified as a high-risk agent.

### Comparing Characteristics of Hospital-Associated Prescriptions With NH-Associated Prescriptions

To align with our aims, we compared hospital-associated prescriptions with NH prescriptions using high-level antibiotic stewardship metrics. For antibiotics with known routes of administration, oral therapies were less prevalent as hospital-associated prescriptions than NH-associated prescriptions (316 of 861 [36.7%] and 172 of 193 [89.1%], *P* < .001). Broad-spectrum coverage for the treatment of SSTIs was often continued from acute care to NHs, as no significant difference was identified between hospital-associated prescriptions and NH-associated prescriptions (29 of 166 [17.5%] and 5 of 28 [17.9%]; *P* = .96). No significant difference in anti-anaerobic prescribing was identified between hospital-associated prescriptions and NH-associated prescriptions (200 of 971 [20.6%] and 34 of 198 [17.2%]; *P* = .27). High-risk, C diffogenic agents were more prevalent as hospital-associated prescriptions than NH-associated prescriptions (440 of 971 [45.3%] and 74 of 198 [37.4%]; *P* = .04). Furthermore, high-risk WHO AWARE antibiotics (watch and reserve agents) were more prevalent as hospital-associated prescriptions than NH-associated prescriptions (644 of 971 [66.3%] and 104 of 198 [52.5%]; *P* < .001).

### Association of Preenrollment Antibiotic Exposure With MDRO Burden in Nursing Homes

Of the 642 patients enrolled, 373 (58.1%) had an antibiotic exposure within 60 days of NH study enrollment; 245 patients (38.2%) had a C diffogenic agent exposure and 279 (43.5%) had a high-risk, WHO AWARE antibiotic exposure (watch or reserve agent) before study enrollment.

**Figure 1.**
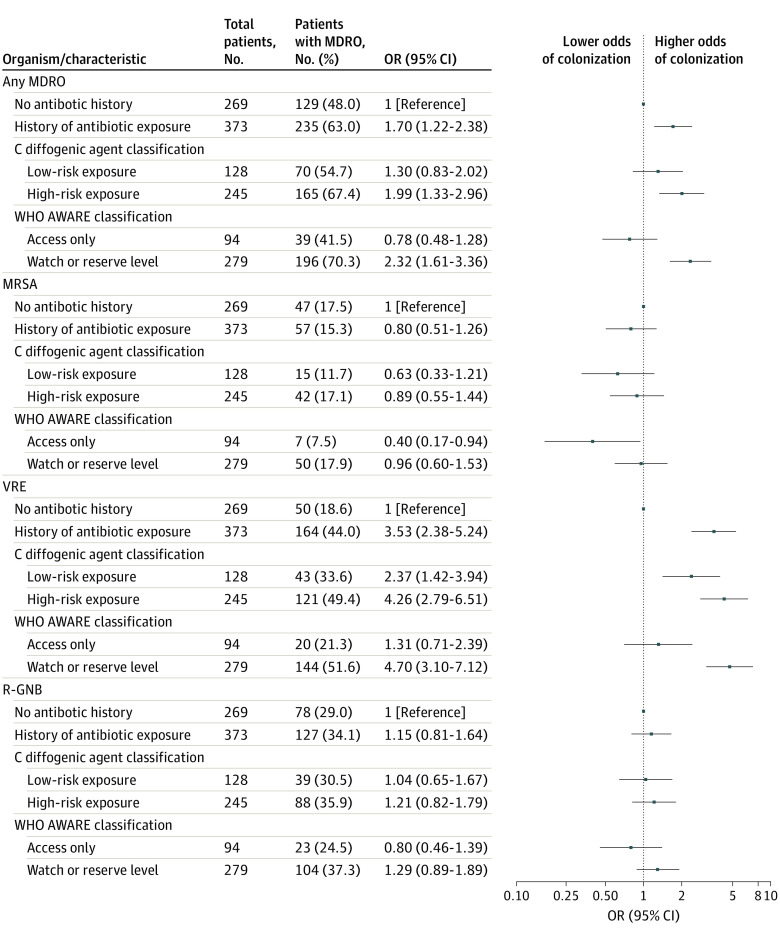
Association of Recent Antibiotic Exposure With Nursing Home Multidrug-Resistant Organism (MDRO) Patient Colonization Information about C diffogenic and World Health Organization (WHO) Access, Watch, and Reserve (AWARE) classifications appears in the Methods section. A description of the multivariable logistic regression model appears in the Statistical Analysis subsection of the Methods section. MRSA indicates methicillin-resistant *Staphylococcus aureus*; R-GNB, resistant gram-negative bacilli; VRE, vancomycin-resistant enterococci.

Overall, 364 patients (56.7%) were colonized with an MDRO on study enrollment: 104 (16.2%) with MRSA; 214 (33.3%) with VRE; and 205 (31.9%) with an R-GNB (Figure 1). In multivariable analysis, preenrollment exposure to antibiotics was associated with MDRO colonization (odds ratio [OR], 1.70; 95% CI, 1.22-2.38) and VRE colonization (OR, 3.53; 95% CI, 2.38-5.24) on comparison with patients without antibiotic exposure histories. In exploratory analysis, the effect size increased on stratification of exposure histories by our high-risk C diffogenic agent classification schema: (MDRO colonization: OR, 1.99; 95% CI, 1.33-2.96; VRE colonization: OR, 4.26; 95% CI, 2.79-6.51). A similar observation was identified when stratifying antibiotic exposure histories by our high-risk, WHO AWARE stewardship classification schema (MDRO colonization: OR, 2.32; 95% CI, 1.61-3.36; and VRE colonization: OR, 4.70; 95% CI, 3.10-7.12).

**Figure 2.**
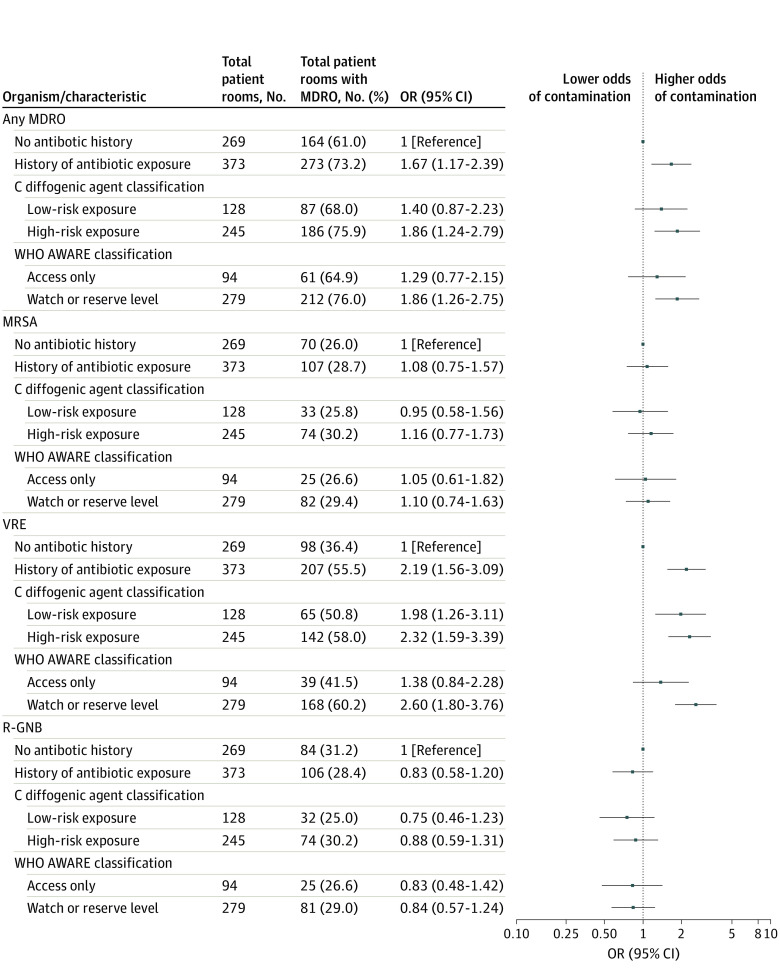
Association of Recent Antibiotic Exposure With Nursing Home Multidrug-Resistant Organism (MDRO) Room Environment Contamination Information about C diffogenic and World Health Organization (WHO) Access, Watch, and Reserve (AWARE) classifications appears in the Methods section. A description of the multivariable logistic regression model appears in the Statistical Analysis subsection of the Methods section. MRSA indicates methicillin-resistant *Staphylococcus aureus*; R-GNB, resistant gram-negative bacilli; VRE, vancomycin-resistant enterococci.

A total of 437 patient room environments (68.1%) were contaminated on study enrollment: 177 (27.6%) with MRSA; 305 (47.5%) with VRE; and 190 (29.6%) with an R-GNB (Figure 2). In multivariable analysis, prestudy enrollment exposure to antibiotics was positively associated with MDRO environmental contamination (OR, 1.67; 95% CI, 1.17-2.39) and VRE environmental contamination (OR, 2.19; 95% CI, 1.56-3.09) on comparison with patients without antibiotic exposure histories. Prestudy enrollment exposure to a C diffogenic agent increased odds of MDRO environmental contamination (OR, 1.86; 95% CI, 1.24-2.79) and VRE environmental contamination (OR, 2.32; 95% CI, 1.59-3.39). Furthermore, prestudy enrollment exposure to a high-risk, WHO AWARE agent significantly increased the odds of MDRO environmental contamination (OR, 1.86; 95% CI, 1.26-2.75) and VRE environmental contamination (OR, 2.60; 95% CI, 1.80-3.76). There were no statistically significant associations between antibiotic exposure and R-GNB outcomes in this patient population. All adjusted ORs and *P *values are reported in eTable 2 in the Supplement.

## Discussion

This study leveraged data collected from a prospective, longitudinal cohort study to characterize antibiotic exposures across the NH patient’s health care continuum (eg, preceding hospital encounter and subsequent NH stay). Our analysis highlighted that antibiotic exposure was common in our postacute care NH patient population. Of particular interest is the observation that most patients with antibiotic exposure were prescribed a high-risk antibiotic, as defined by heightened risk for CDI or the WHO’s AWARE antibiotic stewardship framework. In our population, hospital exposure to high-risk antibiotics was positively associated with baseline NH MDRO outcomes, largely owing to increased risk of VRE colonization and room environment contamination, on comparison with patients without antibiotic exposure histories. Collectively, our results suggest that patients receiving postacute care in NHs are a population with several high-value antibiotic stewardship targets.

Nearly two-thirds of enrolled NH patients had an antibiotic exposure during their health care continuum. In our study, nearly 60% percent of patients received an antibiotic before study enrollment: virtually all (96%) were associated with a prior hospitalization. Antibiotic prescribing at hospital discharge is common and often suboptimal.^41,42^ Given reduced antibiotic stewardship oversight, poor discharge documentation, and frequent empirical and broad-spectrum prescribing practices, antibiotic prescribing on discharge to NH is associated with adverse events, including rehospitalization and CDI.^41,42,43,44^ Alongside the large proportion of patients receiving a hospital-associated prescriptions, nearly one-fifth of enrolled patients received an NH-associated prescription for a new indication during their NH stay, predominantly for UTIs. Previous research demonstrated that NH antibiotic prescribing is often inappropriate, with some estimates surpassing 50%, particularly due to inappropriate urine culturing and treatment of asymptomatic bacteriuria.^5,45,46^ Furthermore, previous work has highlighted the need to study and design high-impact antibiotic stewardship interventions targeting postacute care NH patient populations.^5,47,48^

Postacute care NH patients were often prescribed antibiotics, predisposing them to a heightened risk of CDI or selection for antibiotic resistance. We found that 44% of antibiotics could be classified as high-risk, C diffogenic agents, while 64% could be classified as high-risk antibiotics prone to selection for antibiotic resistance (per the WHO’s AWARE framework). Prestudy enrollment antibiotic exposure, markedly to either of our high-risk antibiotic categories, was positively associated with MDRO colonization and room environment contamination at NH study enrollment. Analysis of individual organisms demonstrated that the association between antibiotic exposure and our composite MDRO outcome was predominantly associated with the antibiotic exposure’s influence on VRE prevalence. These findings support previous research demonstrating that antibiotics have both short- and long-term effects on the intestinal microbiota,^15^ which can lead to increased susceptibility to CDI,^49^ sepsis,^22^ colonization, and intestinal dominance with VRE^17,50^ and patient shedding onto their proximal environment.^19,20,21^ Furthermore, our work complements research illustrating that specific antibiotics may predispose patients to a heightened risk for adverse events, such as patient colonization, shedding, and infection.^19,20,21,23^ Collectively, our observations uniquely link *C difficile* antibiotic stewardship and global antibiotic stewardship metrics with infection prevention efforts in NHs while supporting the emerging hypothesis that previous hospital exposures (ie, antibiotic stewardship practices and MDRO endemicity) have downstream consequences on regional NH MDRO burden.^8,9,10^

These observations underscore the potential value of integrating hospital and NH antibiotic stewardship and infection prevention programming.^8,9,10^ Emerging literature has identified that hospital-initiated NH antibiotic stewardship programming can improve NH antibiotic utilization and urine culturing practices.^51^ For example, a collaborative hospital-initiated, NH antibiotic stewardship intervention identified antibiotic stewardship targets and successfully demonstrated reduced fluoroquinolone (a high-risk C diffogenic agent and WHO watch antibiotic) days of therapy and CDI rates in participating NHs.^51^ Given the emerging interconnectedness between hospitals and NHs^3,4^ and the requirement for NH implementation of antibiotic stewardship programming,^47^ our research emphasizes the potential utility of applying the horizontal and vertical intervention paradigm^52^ to improve regional antibiotic utilization, reduce MDRO transmission, and minimize antibiotic-associated adverse events.

### Limitations

This study has several limitations. First, antibiotic data were abstracted from the patient’s NH charts. We relied on the accuracy of NH documentation and hospital discharge reports from multiple medical records systems, 2 common sources of data inaccuracy, and thus, we can only make general assumptions about the antibiotic indications. Therefore, we used objective and common antibiotic stewardship metrics such as double anaerobic coverage. Second, the completeness of antibiotic duration data varied, including exposures from hospitalizations and NH stays. Thus, we defined antibiotic exposures by location of therapy initiation to minimize facility-associated biases in prescribing and antibiotic stewardship programming. Third, we did not assess the association between recent prestudy enrollment antibiotic exposures and longitudinal NH MDRO outcomes. Tailored cohort studies that comprehensively collect temporal antibiotic exposure data are needed to elucidate the association of antibiotic exposures with longitudinal MDRO outcomes, including patient shedding, new acquisition, and intrafacility transmission. Fourth, we did not perform room environmental surveillance before patient occupancy, and thus, we cannot directly measure patient shedding. The median (IQR) days from NH admission that patients were enrolled at was 6.0 (IQR, 3.0-7.0), plenty of time for shedding to the room environment.^39,53,54^ Fifth, the determination of clinically relevant differences in hospital- and NH-antibiotic prescription practices requires further in-depth study. Sixth, facility variation in infection prevention and antibiotic stewardship efforts must be considered when evaluating the contribution of antibiotic exposures to MDRO epidemiology. Surveys conducted at our study facilities indicate both similarities and differences in infection prevention and cleaning practices.^55,56^ To account for facility-level variations, we adjusted for clustering by facility in our logistic regression models.

## Conclusions

In this study, we found that antibiotic use is common in our predominantly post-acute care NH patient population. Most antibiotics were classified as a high-risk exposure, as defined by heightened risk for CDI or according to the WHO’s AWARE antibiotic stewardship framework. Furthermore, we found that recent exposure to high-risk antibiotics was positively associated with MDRO colonization and room environment contamination at NH study enrollment. These findings highlight the potential importance of strengthening hospital and NH programming to improve antibiotic stewardship and reduce MDRO burden. Future research should elucidate the association of antibiotic exposures across a patient’s health care continuum with longitudinal NH MDRO outcomes. Furthermore, future studies should assess whether hospital-based antibiotic stewardship interventions, such as targeting high-risk antibiotics on discharge, produce beneficial downstream effects on regional MDRO epidemiology.

## Notes

Supplement. **eFigure 1. **Conceptual Diagram of Antibiotic Exposure’s Association With Patient and Room Environment Contamination With Multidrug-Resistant Organisms **eFigure 2.** Schematic for Location of Therapy Initiation Classifications **eTable 1. **High-Risk Antibiotic Classifications for Study Antibiotics **eTable 2.** Association of Recent Antibiotic Exposure With Nursing Home Multidrug-Resistant Organism Burden
