# Supplementary material for: Association of Exposure to High-risk Antibiotics in Acute Care Hospitals With Multidrug-Resistant Organism Burden in Nursing Homes
Source: JAMA Netw Open. 2022 Feb 1;5(2):e2144959. doi: 10.1001/jamanetworkopen.2021.44959 (PMC8808331; doi:10.1001/jamanetworkopen.2021.44959)
Supplement: Supplement. — eFigure 1. Conceptual Diagram of Antibiotic Exposure’s Association With Patient and Room Environment Contamination With Multidrug-Resistant Organisms eFigure 2. Schematic for Location of Therapy Initiation Classifications eTable 1. High-Risk Antibiotic Classifications for Study Antibiotics eTable 2. Association of Recent Antibiotic Exposure With Nursing Home Multidrug-Resistant Organism Burden [file jamanetwopen-e2144959-s001.pdf]

Supplemental Online Content

Gontjes KJ, Gibson KE, Lansing BJ, et al. Association of exposure to high-risk antibiotics in acute care hospitals with multidrug-resistant organism burden in nursing homes. *JAMA Netw Open.* 2022;5(2):e2144959. doi:10.1001/jamanetworkopen.2021.44959

eFigure 1. Conceptual Diagram of Antibiotic Exposure’s Association With Patient and Room Environment Contamination With Multidrug-Resistant Organisms

eFigure 2. Schematic for Location of Therapy Initiation Classifications

eTable 1. High-Risk Antibiotic Classifications for Study Antibiotics

eTable 2. Association of Recent Antibiotic Exposure With Nursing Home Multidrug-Resistant Organism Burden

This supplemental material has been provided by the authors to give readers additional information about their work.

© 2022 Gontjes KJ et al. *JAMA Network Open.*

eFigure 1. Conceptual Diagram of Antibiotic Exposure's Association With Patient and Room Environment Contamination With Multidrug-Resistant Organisms

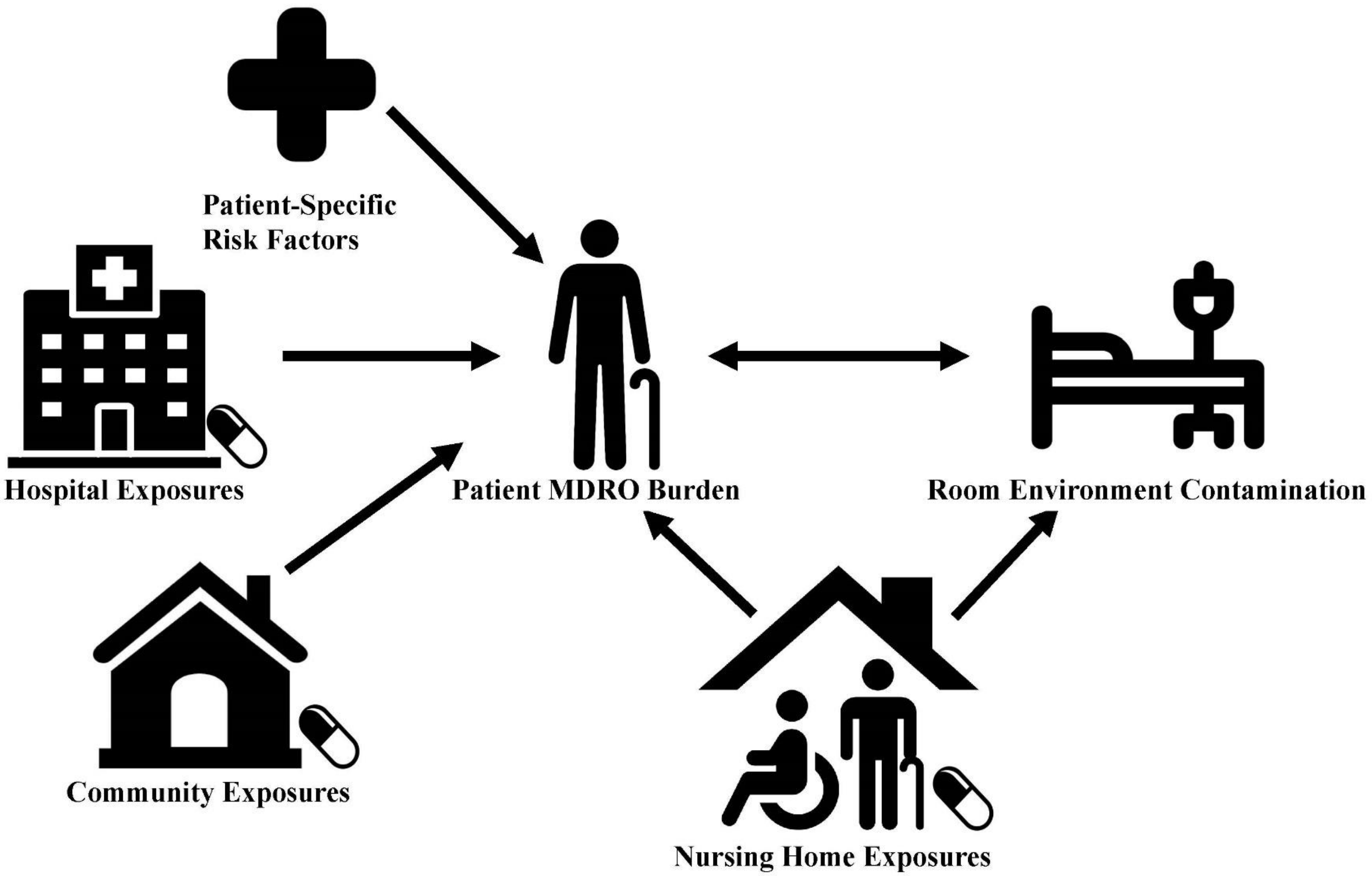

© 2022 Gontjes KJ et al. *JAMA Network Open.*

eFigure 2. Schematic for Location of Therapy Initiation Classifications

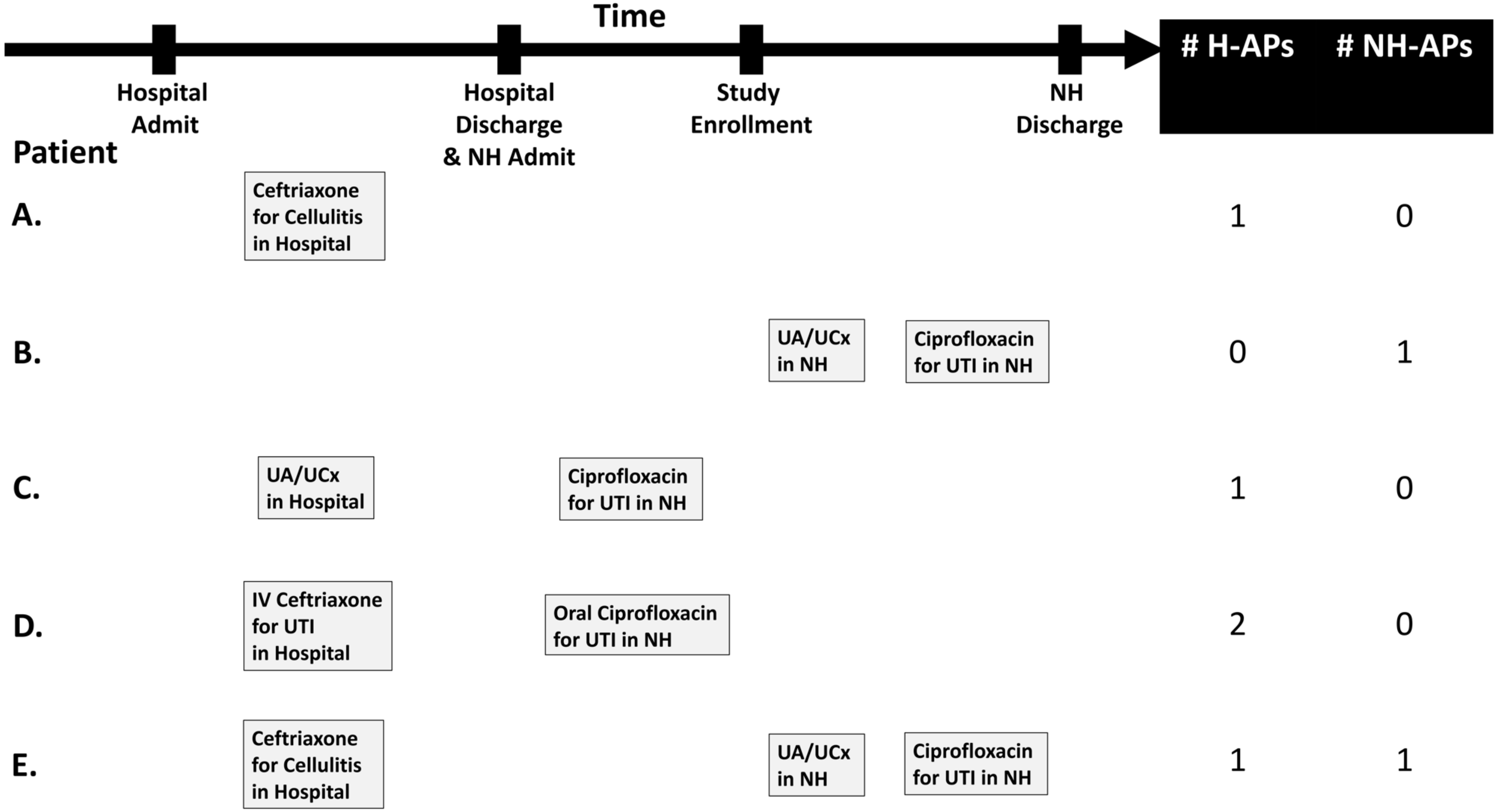

Abbreviations: IV, intravenous; H-AP, hospital-associated prescription; NH, nursing home; NH-AP, nursing home-associated prescription; UA/UCx, urinalysis and urine culture; UTI, urinary tract infection.

Antibiotics were classified as hospital-associated prescriptions if they were associated with indications diagnosed during the patient's hospitalization. NH prescriptions identified as a continuation of hospital therapy or associated with a hospital indication were also classified as hospital-associated prescriptions, unless identified as a novel new start. Therefore, antibiotics were classified as NH-associated prescriptions if they were initiated during the patient's NH stay and were (1) for a new indication; (2) not a continuation of therapy for a prior diagnosis; and/or (3) not an oral transition from a previously intravenous antibiotic. Antibiotics prescribed in alternative locations (ie, emergency department, antecedent NH stay, or outpatient clinics) were classified as other.

© 2022 Gontjes KJ et al. *JAMA Network Open.*

eTable 1. High-Risk Antibiotic Classifications for Study Antibiotics

**Antibiotic Name**	**C. diffogenic Classification[a]**	**WHO AWaRe Classification[b]**
Amoxicillin	Low-Risk	Access
Amoxicillin-Clavulanate	High-Risk	Access
Ampicillin	Low-Risk	Access
Ampicillin-Sulbactam	High-Risk	Access
Azithromycin	Low-Risk	Watch
Aztreonam	Low-Risk	Reserve
Cefazolin	Low-Risk	Access
Cefepime	High-Risk	Watch
Cefoxitin	Low-Risk	Watch
Cefpodoxime	High-Risk	Watch
Ceftaroline	High-Risk	Reserve
Ceftazidime	High-Risk	Watch
Ceftriaxone	High-Risk	Watch
Cefuroxime	Low-Risk	Watch
Cephalexin	Low-Risk	Access
Ciprofloxacin	High-Risk	Watch
Clarithromycin	Low-Risk	Watch
Clindamycin	High-Risk	Access
Daptomycin	Low-Risk	Reserve
Doxycycline	Low-Risk	Access
Ertapenem	High-Risk	Watch
Erythromycin	Low-Risk	Watch
Fidaxomicin	Low-Risk	Watch
Gentamicin	Low-Risk	Access
Imipenem	High-Risk	Watch
Levofloxacin	High-Risk	Watch

© 2022 Gontjes KJ et al. *JAMA Network Open.*

eTable 1. High-Risk Antibiotic Classifications for Study Antibiotics (Continued)

Antibiotic Name	C. diffogenic Classification[a]	WHO AWaRe Classification[b]
Linezolid	Low-Risk	Reserve
Meropenem	High-Risk	Watch
Metronidazole	Low-Risk	Access
Minocycline	Low-Risk	Reserve
Moxifloxacin	High-Risk	Watch
Nafcillin	Low-Risk	Access
Nitrofurantoin	Low-Risk	Access
Penicillin	Low-Risk	Access
Piperacillin-Tazobactam	High-Risk	Watch
Rifampin	Low-Risk	Watch
Rifaximin	Low-Risk	Watch
Sulfamethoxazole-trimethoprim	Low-Risk	Access
Tigecycline	Low-Risk	Reserve
Tobramycin	Low-Risk	Watch
Vancomycin	Low-Risk	Watch

[a] Antibiotics were defined as high-risk, C. diffogenic agents if they predispose patients to a markedly high-risk of *Clostridioides difficile* infection (CDI) as per literature: fluoroquinolones, 3rd/4th generation cephalosporins, penicillin combinations, lincosamides, and carbapenems. Patients without exposure to a high-risk C. diffogenic agent, but with exposure to other antibiotics, were classified as having a low-risk antibiotic exposure. Main text references 22-26 were used to create this high-risk classification scheme.
[b] Antibiotics were further classified by the WHO's 2019 Access, Watch, and Reserve (AWaRe) antibiotic classification framework. See main text references 27-28 for more information on the WHO's AWaRe classification framework.

© 2022 Gontjes KJ et al. *JAMA Network Open.*

eTable 2. Association of Recent Antibiotic Exposure With Nursing Home Multidrug-Resistant Organism Burden

Characteristic	Any MDRO aOR (95% CI)[a]	P-value	MRSA aOR (95% CI)[a]	P-value	VRE aOR (95% CI)[a]	P-value	R-GNB aOR (95% CI)[a]	P-value
**Proximal Outcome:** Patient Colonization								
No Antibiotic Exposure	1.00 (Referent)		1.00 (Referent)		1.00 (Referent)		1.00 (Referent)	
History of Antibiotic Exposure	**1.70 (1.22-2.38)**	**0.002**	0.80 (0.51-1.26)	0.34	**3.53 (2.38-5.24)**	**< 0.001**	1.15 (0.81-1.64)	0.44
**C. diffogenic Agent Classification[b]**								
Low-Risk Exposure History	1.30 (0.83-2.02)	0.25	0.63 (0.33-1.21)	0.17	**2.37 (1.42-3.94)**	**< 0.001**	1.04 (0.65-1.67)	0.88
High Risk Exposure History	**1.99 (1.33-2.96)**	**< 0.001**	0.89 (0.55-1.44)	0.65	**4.26 (2.79-6.51)**	**< 0.001**	1.21 (0.82-1.79)	0.34
**WHO AWaRe Classification[c]**								
Access-Only Exposure History	0.78 (0.48-1.28)	0.33	**0.40 (0.17-0.94)**	**0.035**	1.31 (0.71-2.39)	0.39	0.80 (0.46-1.39)	0.42
Watch/Reserve Exposure History	**2.32 (1.61-3.36)**	**< 0.001**	0.96 (0.60-1.53)	0.86	**4.70 (3.10-7.12)**	**< 0.001**	1.29 (0.89-1.89)	0.18
**Distal Outcome:** Room Environment Contamination								
No Antibiotic Exposure	1.00 (Referent)		1.00 (Referent)		1.00 (Referent)		1.00 (Referent)	
History of Antibiotic Exposure	**1.67 (1.17-2.39)**	**0.005**	1.08 (0.75-1.57)	0.67	**2.19 (1.56-3.09)**	**< 0.001**	0.83 (0.58-1.20)	0.32
**C. diffogenic Agent Classification[b]**								
Low-Risk Exposure History	1.40 (0.87-2.23)	0.16	0.95 (0.58-1.56)	0.84	**1.98 (1.26-3.11)**	**0.003**	0.75 (0.46-1.23)	0.26
High Risk Exposure History	**1.86 (1.24-2.79)**	**0.003**	1.16 (0.77-1.73)	0.47	**2.32 (1.59-3.39)**	**< 0.001**	0.88 (0.59-1.31)	0.52
**WHO AWaRe Classification[c]**								
Access-Only Exposure History	1.29 (0.77-2.15)	0.33	1.05 (0.61-1.82)	0.86	1.38 (0.84-2.28)	0.21	0.83 (0.48-1.42)	0.49
Watch/Reserve Exposure History	**1.86 (1.26-2.75)**	**0.002**	1.10 (0.74-1.63)	0.65	**2.60 (1.80-3.76)**	**< 0.001**	0.84 (0.57-1.24)	0.37

Abbreviations: aOR, adjusted odds ratio; Bold, $p < 0.05$; CI, confidence interval; MDRO, multidrug-resistant organism; MRSA, methicillin-resistant *Staphylococcus aureus*; R-GNB, resistant Gram-negative bacilli; VRE, vancomycin-resistant enterococci.

[a] Multivariable logistic regression model was adjusted for age, sex, race (White vs other), Charlson Comorbidity Index score, Physical Self-Maintenance score, indwelling device (urinary catheter or feeding tube) present on enrollment, hospital stay greater than 14 days, and nursing home days to enrollment. Antibiotic indicator variables were added for baseline antibiotic exposures. The initial

© 2022 Gontjes KJ et al. *JAMA Network Open.*

model used an indicator variable for any antibiotic exposure, while the exploratory model had two indicator variables: low-risk exposure and high-risk antibiotic exposure histories. For each model, the reference group consisted of patients without antibiotic exposure histories. All regression analyses were adjusted for clustering by facility.

[b] Antibiotics were defined as high-risk, C. diffogenic agents if they predispose patients to a markedly high-risk of *Clostridioides difficile* infection (CDI) as per literature: fluoroquinolones, 3rd/4th generation cephalosporins, penicillin combinations, lincosamides, and carbapenems. Patients without exposure to a high-risk C. diffogenic agent, but with exposure to other antibiotics, were classified as having a low-risk antibiotic exposure. References 22-26 were used to create this high-risk classification schema.

[c] Antibiotics were further classified by the WHO's 2019 Access, Watch, and Reserve (AWaRe) antibiotic classification framework. See references 27-28 for more information on the WHO's AWaRe classification framework. Only 22 patients were exposed to a Reserve antibiotic prior to study enrollment. Therefore, we decided to dichotomize the WHO AWaRe categories into a high-risk variable. Briefly, patients with exposure to only Access agents were classified as having a low-risk exposure history, while patients with exposure to Watch or Reserve agents as their highest AWaRe category exposure were classified as having a high-risk WHO AWaRe exposure history.

© 2022 Gontjes KJ et al. *JAMA Network Open.*
